# Unilateral temporomandibular joint disorders diagnosed as both disc displacement without reduction and osteoarthritis show vertical craniofacial asymmetry in women

**DOI:** 10.22514/jofph.2024.029

**Published:** 2024-09-12

**Authors:** Jung Han, Sung-Hwan Choi, Hyung Joon Ahn, Jeong-Seung Kwon, Younjung Park, Yoon Jeong Choi

**Affiliations:** ^1^Department of Orthodontics, Institute of Craniofacial Deformity, Yonsei University College of Dentistry, 03722 Seoul, Republic of Korea; ^2^Department of Orofacial Pain and Oral Medicine, Yonsei University College of Dentistry, 03722 Seoul, Republic of Korea

**Keywords:** Temporomandibular joint disorder, Facial asymmetry, Unilateral disorder, Disc discplacement, Osteoarthritis

## Abstract

This study investigated the effects of unilateral temporomandibular joint disorders (TMJDs), specifically disc displacement without reduction and osteoarthritis on one side of the temporomandibular joint (TMJ), on facial asymmetry in women, while the contralateral TMJ exhibits normal findings. Participants were retrospectively enrolled and divided into an affected group (n = 42 with unilateral TMJD) and a control group (n = 49 with bilateral healthy TMJs). The affected group was dagnosed with osteoarthritis on cone-bema computed tomograph and anterior disk displacement without reduction on magnetic resonance imaging. The control group showed normal findings bilaterally on both tests. Facial asymmetry was quantified using an asymmetry index derived from posteroanterior cephalograms, comparing both groups. The relationship between TMJD sub-findings and facial asymmetry was also investigated. Significant increases in the asymmetry indexes of the vertical distances from the antegonial notch and gonion to a horizontal reference plane were observed in the affected group (*p* < 0.05). Additionally, there was a noticeable upward canting of the maxillary, occlusal, and mandibular planes towards the affected side (*p* < 0.05). Horizontal asymmetry did not differ significantly between groups (*p* > 0.05). Parafunctional habits in the affected group were correlated with higher asymmetry indexes of the antegonial notch distance (*p* < 0.05). Women with unilateral TMJD exhibit significantly greater vertical facial asymmetry compared to those without TMJD. These findings may assist clinicians in diagnosing vertical asymmetry in patients with unilateral TMJD using cephalograms and in predicting facial asymmetry progression.

## 1. Introduction

Temporomandibular disorders, which include muscle and joint disorders, may cause not only functional problems but also structural problems in craniofacial morphology. Internal derangement, one of the most common temporomandibular joint disorders (TMJDs), has been reported to affect mandibular morphology [1]. With anterior displacement and subsequent disc deformation, the articular eminence would be flattened, and the condylar surface would undergo degenerative changes over time [2]. In the case of unilateral disc displacement (DD), the ramus height may become shorter on the affected side than on the other unaffected side, leading to menton deviation to the affected side, although the causality remains largely unclarified [1, 3, 4]. Consequently, as the degree of DD gets severe, canting of the mandibular and occlusal planes may get severe [5]. To observe internal derangement, magnetic resonance imaging (MRI) has been widely used due to its superiority in detecting soft tissue pathology [6, 7, 8].

Osteoarthritis (OA), another common TMJD, is also known to affect facial morphology [8]. OA refers to a destructive process in which the articular surfaces of the condyle and fossa change [9]. Articular tissues of the joint soften with OA when excessive force is applied, resulting in dysfunctional remodeling in which the bony surface in contact with the joint surface is resorbed [10]. As the condylar volume decreases, ramus height decreases, followed by mandibular retrusion [11]. Furthermore, in patients with OA in unilateral TMJ, the ramus height of the affected side may become smaller than that of the unaffected side, and the mandibular midline may deviate to the affected side [8], similar to unilateral internal derangement. The role of inflammation on condylar asymmetry during growing especially in girls has been reported in patients having juvenile idiopathic arthritis [12]. Cone-beam computed tomography (CBCT) is more appropriate to diagnose OA than MRI because of its superiority in evaluating cortical bone integrity and osseous abnormalities [13, 14].

Previous studies have investigated the relationship between the presence of unilateral TMJD and facial asymmetry [15]. However, the definition and scope of TMJDs vary in each study. Most of them have defined TMJD as internal derangement. Nevertheless, some investigations lacked validity due to the absence of MRI in evaluating internal derangement [4]. The categorization of TMJD into three to five subsets, based on unilateral versus bilateral internal derangement and DD with or without reduction, has led to controversial findings [3, 16]. Despite this, there is a general consensus that more severe TMJ degeneration on one side is associated with facial asymmetry. Given that the condition was more advanced in cases of DD without reduction than in those of DD with reduction, it proposes a targeted comparison between individuals with both normal TMJ and those with unilateral DD without reduction. This approach aims to clarify the impact of advanced TMJD on facial asymmetry by contrasting these two distinct conditions.

Meanwhile, a limited number of studies have classified TMJD based on OA findings of the condyles, such as erosion, osteophytes, or flattening. They suggested that unilateral OA resulted in horizontal discrepancy such as menton deviation, but the influence of unilateral OA on the vertical discrepancy of facial asymmetry remains controversial [8, 17]. Although the exact relationship between DD without reduction and OA has been barely clarified, DD is known to be an important factor affecting OA [18]. Therefore, for a comprehensive understanding of the impact of anatomical pathologic conditions in the TMJ on facial morphology, particularly asymmetry, it is essential to include patients with both DD and OA in the research scope. The combined impact of unilateral TMJD, including both DD without reduction and OA, on facial asymmetry has not been extensively studied. Furthermore, given the condyle is crucial in the growth and development of the mandible, the impact of unilateral pathologic condition on TMJ to facial asymmetry needs to be clarified [12]. Therefore, obtaining more precise and consistent results necessitates a precise definition of unilateral TMJD that includes both DD without reduction and OA.

This study aimed to investigate the effect of unilateral TMJD, diagnosed as DD without reduction and OA on only one side of TMJ but normal findings on the other side of TMJ, on facial asymmetry using a posteroanterior (PA) cephalogram, which can simply show differences between the affected and unaffected sides. This study was performed by comparing the PA measurements between the TMJD and normal side and the asymmetry between the unilateral TMJD and control groups.

## 2. Materials and methods

### 2.1 Patients

This study was retrospectively conducted on patients who visited the Department of Orofacial Pain & Oral Medicine, Yonsei University Dental Hospital, from January 2019 to June 2022. These patients had undergone PA cephalography, MRI, and CBCT within a 6-month period for the diagnostic evaluation of TMJ discomfort (Fig. 1). MRI and CBCT scans were performed on these patients presenting subjective TMJ discomforts to refine the diagnostic process.

**Fig. 1.** Flow chart of patient selection. PA, posteroanterior; CBCT, cone-beam computed tomography; MRI, magnetic resonance imaging; OA, osteoarthritis; TMJ, temporomandibular joint.

The patients were diagnosed according to the diagnostic criteria for TMJD [19]. The affected group comprised patients who presented with both DD without reduction and OA in one TMJ, while the other TMJ exhibited normal findings. The control group included patients who were confirmed to have normal findings in both TMJs but experienced subjective discomfort (Fig. 2). Specifically, patients demonstrating structural changes on MRI and CBCT scans were classified as the affected group, whereas those with acute symptoms but no structural changes were categorized as the control group. PA cephalograms used in this study were obtained using a Rayscan machine (RAYFace, Ray Co. Ltd., Hwaseong, Korea) with a tube voltage of 90 kVp and a tube current of 13 mA and collected from the picture archiving and communication system of the Yonsei University Dental Hospital as JPEG files. The images had a resolution of 1930 × 2238 pixels and a pixel spacing of 0.13 mm. Each pixel was represented by a single grayscale channel with values ranging from 0–255. For diagnosing DD without reduction, MRI of the TMJ was conducted using a 3.0 T scanner (Pioneer; GE Healthcare, Waukesha, WI, USA) with 16-channel flex large coil. A sagittal section view, perpendicular to the long axis of the condylar head with a slice thickness of 2.5 mm, was obtained using proton density-weighted and T2 weighted sequences in the closed-mouth and open-mouth positions. For diagnosing OA, CBCT images were obtained with an Alphard 3030 instrument (Asahi Roentgen Industries, Kyoto, Japan) using the following parameters: tube voltage, 80 kVp; tube current, 8 mA; exposure time, 17 seconds; and field of view, 154 × 154 mm. Image reconstruction was performed to obtain a true sagittal and coronal TMJ view with a slice thickness of 1.0 mm according to the long axis of the condylar head by a skilled radiographer using OnDemand 3D software (version 1.0, Cybermed, Seoul, Korea). OA was diagnosed when features like subchondral cyst, erosion, generalized sclerosis, or osteophyte were observed on the condyle [19]. For evaluation of these images, two experienced oral and maxillofacial radiologists evaluated the CBCT and MRI images, resolving any disagreements by consensus.

**Fig. 2.** TMJ radiographs comparing affected and control groups. In the affected group, TMJ radiographs show a displaced disc at the closed jaw position (indicated by a solid arrow) on the affected side, which does not reduce at the open position, indicating disc displacement without reduction. The unaffected side in the affected group and the control group show normal disc positioning above the mandibular condyle in both closed and open positions. Additionally, the affected side in the affected group demonstrates osteophyte formation (dotted arrow), erosion, and generalized sclerosis (arrowheads), indicative of osteoarthritis (OA) [19]. Conversely, the unaffected side in the affected group shows flattening but retains cortical integrity, suggesting no active inflammation. The control group displays normal condyle morphology. TMJ, temporomandibular joint; MRI, magnetic resonance imaging; CBCT, cone-beam computed tomography.

Initially, 61 and 119 patients in the affected and control groups, respectively, were enrolled from archives based on electronic medical records. Thereafter, patients were selected based on the following inclusion and exclusion criteria. The inclusion criteria were women patients, excluding sex differences, whereas the exclusion criteria were history of orthodontic treatment or craniofacial surgery, synovial chondromatosis, adhesion, low-quality images, and >6-month interval among MRI, CBCT and PA cephalogram. From the chart review, we recorded the age and five TMJD sub-findings: limitation of mouth opening, subjective pain, familiar pain on palpation, parafunctional habit, and unilateral chewing. Each TMJD sub-finding was labeled as 1 if present and 0 if absent, based on the following definitions:

• Limitation of mouth opening: Inability to open the mouth to 40 mm.

• Subjective pain: Self-reported pain in the TMJ area.

• Familiar pain on palpation: Reproduction of familiar pain upon palpation of the TMJ.

• Parafunctional habits: Non-functional jaw activities during both awake and asleep states.

• Unilateral chewing: Predominantly chewing on one side.

For the calculation of the minimum sample size, the effect size was first determined based on the vertical facial height as the primary endpoint, using data from a previous study [4]. The minimal sample size was calculated to be 37 patients for each group using the G-power program (G*Power 3.1, University of Düsseldorf, Düsseldorf, Germany) with a significance level of a *p*-value less than 0.05, a power of 80%, and an effect size of 0.8. Finally, 42 patients in the affected group and 49 in the control group were included in this study (Fig. 1).

### 2.2 Measurements

Facial asymmetry was measured using PA cephalograms. For convenience, the PA cephalograms were flipped horizontally to allow the affected side to be located on the right side. In the control group, PA cephalograms with the menton (Me) on the right side were set as the standard, and those with the Me on the left side were flipped horizontally.

We defined the horizontal and vertical reference planes (HRP and VRP, respectively) and performed seven linear and six angular measurements using the reference planes and four landmarks of condylion (Co), antegonion (Ag), gonion (Go), and Me (Table 1 and Fig. 3): for the linear parameters, (1) VRP-Co, (2) VRP-Ag, (3) VRP-Me, (4) HRP-Ag, (5) HRP-Go, (6) Co-Ag and (7) Co-Me; for the angular parameters (a) HRP-J angle, (b) HRP-U6 angle, (c) HRP-Ag angle, (d) VRP-Co•Ag angle, (e) VRP-Me angle and (f) Co-Ag-Me angle. Positive values of (a), (b) and (c) indicated that the left sides were below the right sides.

**Table 1.** Linear and angular measurements on posteroanterior cephalogram.

Linear and angular measurements		
Reference planes and landmarks		
	Horizontal reference plane (HRP)	A line connecting the left and right intersection points between the superior border of the greater wing of the sphenoid bone and the lateral margin of the orbital wall
	Vertical reference plane (VRP)	A line connecting anterior nasal spine (ANS) and the midpoint of the HRP
	Condylion (Co)	The most superior point of the condyle
	Antegonion (Ag)	The deepest point in the curvature of the antegonial notch
	Gonion (Go)	The most lateral point of the mandibular angle
	Menton (Me)	The lowest point on the symphyseal shadow of the mandible
Linear parameters		
	VRP-Co	The perpendicular distance of Co to VRP
	VRP-Ag	The perpendicular distance of Ag to VRP
	VRP-Me	The perpendicular distance of Me to VRP, whose value is positive when Me is on the right side
	HRP-Ag	The perpendicular distance of Ag to HRP
	HRP-Go	The perpendicular distance of Go to HRP
	Co-Ag	A distance of Ag to Co
	Co-Me	A distance of Me to Co
Angular parameters		
	HRP-J angle	The angle between HRP and a line connecting right and left jugular (J) points, which value is positive when the left side is below than the right side
	HRP-U6 angle	The angle between HRP and a line connecting the right and left maxillary first molars (UR6 and UL6, respectively), which value is positive when the left side is below than the right side
	HRP-Ag angle	The angle between HRP and a line connecting the right and left Ag, which value is positive when the left side is below than the right side
	VRP-Co•Ag angle	The angle between VRP and a line connecting Co and Ag, which value is positive when Co is farther from VRP than Ag
	VRP-Me angle	The angle between VRP and a line connecting ANS and Me, which value is positive when Me is on the right side
	Co-Ag-Me angle	The angle between Co, Ag, and Me

*UR6 and UL6, the maxillary right and left first molar, respectively.*

**Fig. 3.** Measurements and reference planes on posteroanterior cephalogram. (A) Reference planes and landmarks; (B) linear measurements: (1) VRP-Co, (2) VRP-Ag, (3) VRP-Me, (4) HRP-Ag, (5) HRP-Go, (6) Co-Ag, and (7) Co-Me; (C) angular parameters: (a) HRP-J angle, (b) HRP-U6 angle, (c) HRP-Ag angle, (d) VRP-Co•Ag angle, (e) VRP-Me angle and (f) Co-Ag-Me angle. (a), (b) and (c) are positive when the left sides are below than the right sides. VRP, Vertical reference plane; Co, Condylion; Ag, Antegonion; Me, Menton; HRP, Horizontal reference plane; Go, Gonion; U6, the maxillary first molar. Please refer to Table 1 for the abbreviations and definitions.

The bilateral parameters, including VRP-Co, VRP-Ag, HRP-Ag, HRP-Go, Co-Ag and Co-Me as linear parameters and VRP-Co•Ag and Co-Ag-Me angles as angular parameters, were converted to an asymmetry index. It was defined as the value obtained by subtracting the right measurements from the left measurements. In the control group, the asymmetry index was converted into an absolute value because both TMJs were normal. All the other parameters were measured unilaterally. The measurements were initially performed in pixel units using PA cephalograms and converted to millimeter (mm) units at a ratio of 70 pixels to 9 mm.

Additionally, to analyze the effect of sagittal and vertical cephalometric patterns on facial asymmetry, raw Digital Imaging and Communications in Medicine CBCT images were converted to lateral cephalometric images using computer software (ZeTTA PACS, version 2.0.3.5, TaeYoung Soft. Co., Seoul, Korea). Lateral cephalometric tracing was performed to measure ANB (A point-Nasion-B point) and Frankfurt-mandibular plane angle (FMA) to evaluate sagittal and vertical cephalometric patterns, respectively, using V-ceph software (version 5.0, Osstem Implant Co., Seoul, Korea). The ANB angle indicates the relative position of the maxilla to the mandible, helping to determine the anteroposterior jaw relationship. The FMA reflects the inclination of the mandibular plane to the base of the skull, indicating the vertical growth pattern of the jaw.

### 2.3 Statistical analysis

Tracing and measurement of PA cephalograms were performed by a single examiner. To evaluate intra-examiner reliability, the intraclass correlation coefficients (ICC) were calculated by comparing the original measurements with the second measurements, performed 1 month later by randomly selecting 10 patients in the affected group and 10 in the control group. The ICC was >0.94, indicating excellent intra-examiner reliability.

Independent *t*-tests were performed to compare asymmetry indexes between the affected and control groups. Independent *t*-tests were also used to compare the asymmetry index between patients with and without the five TMJD sub-findings in the affected group. All statistical analyses were performed using Statistical Package for the Social Sciences software version 25 (SPSS, IBM Corp., Chicago, IL, USA).

## 3. Results

The patient demographic characteristics are presented in Table 2. The affected group, with a mean age of 46.7 ± 16.4 years, was significantly older than the control group, with a mean age of 38.1 ± 15.7 years (*p *= 0.012). In the sagittal and vertical skeletal analyses, there were no significant differences in ANB and FMA between the two groups (*p *= 0.062), and both groups showed normal sagittal and vertical skeletal relationships. The number of patients presenting the five TMJD sub-findings in each group was also described (Table 2).

**Table 2.** Demographic features of patients.

		Affected group (n = 42)	Control group (n = 49)	*p *value
Demographic features				
Age (yr)		46.7 ± 16.4	38.1 ± 15.7	0.012*
Pain duration (mon)		38.1 ± 26.4	4.3 ± 4.6	<0.001***
ANB (º)		4.2 ± 1.8	3.3 ± 2.3	0.062
FMA (º)		23.2 ± 5.6	24.6 ± 5.7	0.248
TMJD sub-findings (number of patients)				
	Limitation of mouth opening	15	7	
	Subjective pain	36	43	
	Familiar pain on palpation	38	43	
	Parafunctional habits	30	40	
	Unilateral chewing	16	31	

ANB, angle formed point A-Nasion-point B; FMA, Frankfort-mandibular plane angle; TMJD, temporomandibular joint disorder. Independent t-tests were performed to compare the demographic features between two groups. *p < 0.05; ***p < 0.001.

Among the linear parameters, HRP-Ag and HRP-Go showed significantly higher facial asymmetry indexes in the affected group than in the control group (*p* = 0.001 and < 0.001, respectively), indicating that the affected group had more severe skeletal vertical discrepancy between the right and left sides than the control group (Table 3). Among the angular parameters, HRP-J, HRP-U6 and HRP-Ag angles were significantly greater in the affected group than in the control group (*p* = 0.014, 0.001 and < 0.001, respectively). The other linear parameters, such as VRP-Ag, VRP-Co, VRP-Me, Co-Ag and Co-Me, and angular parameters, such as VRP-Co•Ag, VRP-Me, and Co-Ag-Me, were not significantly different between the affected group and control group (*p* > 0.05, Fig. 4).

**Table 3.** Comparison of asymmetry index between affected and control groups.

		Affected group (n = 42)	Control group (n = 49)	*p *value
Linear parameters				
	VRP-Ag	−2.6 ± 3.3	−1.5 ± 2.4	0.671
	VRP-Co	0.2 ± 3.2	−0.0 ± 3.2	0.756
	VRP-Meª (mm)	2.6 ± 2.1	2.4 ± 1.1	0.671
	HRP-Ag	2.9 ± 2.7	1.2 ± 1.9	0.001**
	HRP-Go	3.3 ± 3.0	1.1 ± 2.6	<0.001*
	Co-Ag	1.9 ± 2.8	1.1 ± 2.4	0.154
	Co-Me	1.4 ± 2.7	2.1 ± 3.0	0.243
Angular parameters				
	HRP-J angle^b^ (º)	0.9 ± 1.3	0.3 ± 1.3	0.014*
	HRP-U6 angle^b^ (º)	1.5 ± 1.2	0.7 ± 1.2	0.001**
	HRP-Ag angle^b^ (º)	2.2 ± 1.6	0.8 ± 1.2	<0.001**
	VRP-Co•Ag angle (º)	1.7 ± 3.1	1.2 ± 3.4	0.449
	VRP-Me angle^b^ (º)	2.6 ± 2.2	2.3 ± 0.9	0.358
	Co-Ag-Me angle (º)	−1.9 ± 3.0	−1.2 ± 4.1	0.332

Independent t test was used. *p < 0.05, **p < 0.01. ^a^Unilateral measurements (asymmetry index not applicable); the mean distance (mm), positive if menton is on the right. ^b^Unilateral measurements (asymmetry index not applicable); the mean angle (º), positive if the left side is lower than the right side or if the menton is on the right. Please refer to Table 1 and Fig. 3 for details on the landmarks and measurements. VRP, Vertical reference plane; HRP, Horizontal reference plane; Ag, Antegonion; Co, Condylion; Me, Menton; Go, Gonion; U6, the maxillary first molar.

**Fig. 4.** Posteroanterior cephalograms of the affected group. Note the significant differences in the vertical distances from the antegonial notch and gonion (Go) to the horizontal reference plane (HRP) between the left and right sides. Additionally, there was a noticeable upward canting of the maxillary, occlusal, and mandibular planes towards the affected side. Please refer to Table 1 and Fig. 3 for details on the landmarks and measurements. Ag, Antegonion; Co, Condylion; Me, Menton; ANS, Anterior nasal spine; Go, Gonion; UR6 and UL6, the maxillary right and left first molar, respectively.

In the affected group, to determine the effect of TMJD sub-findings on facial asymmetry, HRP-Ag and HRP-U6 angles, which represent skeletal and dental vertical discrepancies, respectively, were selected and compared between patients depending on the presence of the five TMJD sub-findings: limited mouth opening, subjective pain, familiar pain on palpation, parafunctional habits, and unilateral chewing (Table 4). Patients with parafunctional habits had significantly higher asymmetry indexes of HRP-Ag, implying that patients with parafunctional habits showed more severe skeletal vertical discrepancies between the right and left sides than those without parafunctional habits (*p* = 0.003).

**Table 4.** Comparison of asymmetry index between patients with and without specific TMJD sub-findings in the affected group.

	With limitation (n = 15)	Without limitation (n = 27)	*p *value
HRP-Ag (mm)	3.1 ± 3.0	2.8 ± 2.6	0.755
HRP-U6 angle (º)	1.4 ± 1.2	1.5 ± 1.3	0.774
	With parafunctional habits (n = 30)	Without parafunctional habits (n = 12)	*p *value
HRP-Ag (mm)	3.7 ± 2.6	1.0 ± 2.2	0.003**
HRP-U6 angle (º)	1.5 ± 1.2	1.5 ± 1.4	0.990
	With unilateral chewing (n = 16)	Without unilateral chewing (n = 26)	*p *value
HRP-Ag (mm)	3.0 ± 3.3	2.8 ± 2.4	0.823
HRP-U6 angle (º)	1.6 ± 1.3	1.5 ± 1.2	0.810

Independent t test was used. *p < 0.05, **p < 0.01. Patients with parafunctional habits in the affected group showed more severe skeletal vertical discrepancies (HRP-Ag) between the right and left sides compared to those without parafunctional habits. However, other variables, particularly unilateral chewing, which is a severe functional asymmetry, might be influenced by the distribution of patients, even though the data presented a normal distribution. Please refer to Table 1 and Fig. 3 for details on the landmarks and measurements. HRP, Horizontal reference plane; Ag, Antegonion; U6, the maxillary first molar.

## 4. Discussion

This study aimed to determine the relationship between unilateral TMJD, defined as having both DD without reduction and OA confirmed using MRI and CT, respectively, and facial asymmetry using PA cephalograms. Patients with unilateral TMJD showed greater vertical height discrepancies and canting of the maxillary, occlusal, and mandibular planes. However, no horizontal discrepancy was observed between the two groups, indicating that unilateral TMJD had no significant relationship with horizontal facial asymmetry. Representative features of the PA cephalogram are shown in Fig. 4. Additionally, in patients with unilateral TMJD, parafunctional habits, such as sleep/awake bruxism, worsen the vertical facial height discrepancy.

In patients with unilateral TMJD in this study, the difference in the vertical dimensions between the right and left sides was remarkable, although there were no differences in the horizontal dimensions, such as VRP-Ag and VRP-Me, which are different findings from those of previous studies. This study included patients with OA and DD without reduction in the TMJD diagnosis. Pathologic changes in the TMJ area due to OA, such as bone resorption of the upper condylar surface and shallowing of the articular fossa, ultimately cause a decrease in the vertical dimension of the TMJ area [11]. In addition, as the articular disc flattens and the superior joint space is reduced, the vertical discrepancy of the right and left facial height becomes prominent in the affected group. For horizontal asymmetry, although the difference between the two groups was not significant, both groups showed a menton deviation >2 mm. Although the patients in the control group in this study were not diagnosed with DD or OA, they might have natural facial asymmetry caused by other factors, such as unilateral crossbite [20], parafunctional habits, and congenital TMJ morphology [21]. Therefore, the horizontal discrepancy between patients with TMJD and healthy individuals was not noticeable compared with the vertical discrepancy.

Among the two different features of TMJD, OA is likely the factor that has more influence on vertical facial asymmetry because the superior bony surface of the condyle can be directly resorbed due to OA [10]. Meanwhile, it is interesting that there was no difference of the asymmetry index in ramus height between the two groups, although there was a significant difference of that in total facial height relative to the horizontal reference plane. Moreover, not only the mandibular plane but also the maxillary and occlusal planes showed significant canting toward the affected side. Presumably, unilateral TMJD caused a reduction in the vertical facial height on the affected side over a long period of time.

In the affected group, patients with parafunctional habits exhibited severe vertical discrepancies. Sleep/awake bruxism is a common parafunctional habit known to affect TMJD [22, 23]. Several studies have reported that muscle hyperactivity of the masseter might cause osseous changes in the condylar surface of the joints [24], contributing to OA [25]. Therefore, parafunctional habits might aggravate vertical facial asymmetry in the affected group.

This study included patients with significantly advanced unilateral TMJD with DD without reduction and condylar OA in only one TMJ. However, facial asymmetry, with differences <2 mm in the vertical dimension and 2º in the canting angle, was statistically significant but clinically insignificant compared with normal participants. The main reasons include the chronic features of TMJD and limitations in sample selection. If patients in the affected group had chronic DD and OA for a long time, they would have had host adaptability [10], which could minimize asymmetric changes. Additionally, as masticatory muscle imbalances can affect asymmetry [26], different results may have been obtained if the participants were divided according to their clinical symptoms. The control group in the present study was classified as the TMJD group in a previous study. Almăşan *et al*. [4] classified patients with unilateral TMJD into the experimental group based on clinical symptoms and reported that there were differences in asymmetric patterns between the experimental and control groups. In this study, the decision to classify individuals with normal imaging findings as the control group, rather than those without symptoms, is based on the self-limiting nature of TMJD symptoms after adequate treatment and the significant effect of anatomical pathologies on facial asymmetry. A comparison between patients with unilateral TMJD and healthy individuals without TMJD symptoms in future studies could reveal a more evident influence on facial asymmetry.

To the best of our knowledge, this is the first study to evaluate the effect of unilateral TMJD with DD without reduction and OA on facial asymmetry. Nevertheless, this study had some limitations. First, the unaffected condyles in the affected group may not have been completely healthy. This was particularly the case when the participants were not diagnosed with OA but considered indeterminate findings due to the intact cortical layer of the condyle [19]. In addition, there is a possibility of underlying diseases in the control group, which are difficult to diagnose using MRI or CBCT because they have several TMJD symptoms. Second, the sample in this study included only female patients, considering the higher prevalence of TMJD in women than in men [27] and the differences in linear TMJ parameters between sexes, which could influence the statistical outcomes. Future research might explore these variations more comprehensively, potentially revealing greater facial asymmetry in mixed-sex samples. Third, it is difficult to diagnose whether the asymmetries were structural, caused by the morphology of the mandible, or positional, resulting from the displacement of the mandible. Last, this study utilized PA cephalograms for assessing facial asymmetry, focusing on two-dimensional differences in frontal images. This perspective was chosen for its prominence in initial clinical assessments of facial asymmetry. Though a three-dimensional analysis, including aspects like condylar volume and yaw rotation, offers a more comprehensive view, PA cephalograms provide a practical alternative for clinicians [28]. This method facilitates the diagnosis and confirmation of vertical asymmetry in unilateral TMJD patients, without requiring complex three-dimensional measurements. In clinical settings, evaluations of facial features are primarily conducted from a frontal perspective, rather than from inferior or lateral viewpoints, rendering two-dimensional assessments highly useful for practical insights. It is effective for early detection and prediction of asymmetry progression, while acknowledging the need for further research integrating three-dimensional analyses for more comprehensive understanding of mandibular asymmetry. Moreover, investigating the effects of unilateral posterior crossbite on the masticatory function of the growing condyle is essential. These studies should also include comparisons with asymptomatic healthy individuals as a control group.

## 5. Conclusions

The findings of this study indicate that patients with unilateral TMJD with both DD without reduction and OA on one side of TMJ might exhibit vertical facial asymmetry. This asymmetry is characterized by shortened facial height and upward canting of the affected side. However, no significant differences were observed in horizontal discrepancies, such as menton deviation, between the affected and control groups. Moreover, the presence of parafunctional habits in the affected group may have exacerbated the vertical facial asymmetry. These results may help clinicians diagnose vertical asymmetry in patients with unilateral TMJD using cephalogram and predict the progression of facial asymmetry.
